# Tumor Necrosis Factor-α and Muc2 Mucin Play Major Roles in Disease Onset and Progression in Dextran Sodium Sulphate-Induced Colitis

**DOI:** 10.1371/journal.pone.0025058

**Published:** 2011-09-19

**Authors:** Poonam Dharmani, Pearl Leung, Kris Chadee

**Affiliations:** Department of Microbiology, Immunology and Infectious Diseases, Gastrointestinal Research Group, Health Sciences Centre, University of Calgary, Calgary, Alberta, Canada; Weizmann Institute of Science, Israel

## Abstract

The sequential events and the inflammatory mediators that characterize disease onset and progression of ulcerative colitis (UC) are not well known. In this study, we evaluated the early pathologic events in the pathogenesis of colonic ulcers in rats treated with dextran sodium sulfate (DSS). Following a lag phase, day 5 of DSS treatment was found clinically most critical as disease activity index (DAI) exhibited an exponential rise with severe weight loss and rectal bleeding. Surprisingly, on days 1-2, colonic TNF-α expression (70-80-fold) and tissue protein (50-fold) were increased, whereas IL-1β only increased on days 7-9 (60-90-fold). Days 3-6 of DSS treatment were characterized by a prominent down regulation in the expression of regulatory cytokines (40-fold for IL-10 and TGFβ) and mucin genes (15-18 fold for Muc2 and Muc3) concomitant with depletion of goblet cell and adherent mucin. Remarkably, treatment with TNF-α neutralizing antibody markedly altered DSS injury with reduced DAI, restoration of the adherent and goblet cell mucin and IL-1β and mucin gene expression. We conclude that early onset colitis is dependent on TNF-α that preceded depletion of adherent and goblet cell mucin prior to epithelial cell damage and these biomarkers can be used as therapeutic targets for UC.

## Introduction

Inflammatory bowel disease (IBD), an umbrella term that includes Crohn's disease and ulcerative colitis (UC), are chronic relapsing inflammatory disorders of the gut that are believed to occur in genetically predisposed individuals due to exposure of unknown environmental and microbial agents [1]. A normal healthy intestine exhibits homeostasis where the mucosal immune system escalates an immune response against pathogens but remains tolerant to antigens derived from food and commensal microbes. Loss of mucosal tolerance is due to an uncontrolled inflammatory cascade resulting from a number of mutual and probably sequential events involving both immune (gut associated lymphoid tissues, GALT and professional antigen presenting cells, APC) and non-immune cells/molecules (epithelial cells of gut and resident microflora) [1], [2]. However, the precise etiology of the pathogenesis of UC is still not known.

To date, studies to unravel the pathogenesis of UC have been focused on various mucosal models of inflammation that closely resembles human colitis. One of the most comprehensively illustrated models of experimental colitis is Dextran Sodium Sulphate (DSS) induced colitis which mimics the clinical and histological features of human UC as the colonic lesions exhibits high homogeneity and reproducibility [3]. Acute and chronic colitis induced by DSS has been used to study changes in metabolically labeled and tissue mucin content [4] and/or changes in epithelial permeability, MPO and pro-inflammatory cytokines [4]. However, the inflammatory mediators that play a role in disease onset and progression of colitis are poorly defined. Clinical and experimental studies using DSS models of colitis suggests that the key contributors in disease pathogenesis include: (i) an alteration in the mucosal barrier integrity and function; (ii) reallocation in the role of pathogen recognition receptors (PRRs) of APCs and, (iii) an immune response skewed towards effector cell function (Th1 and probably Th2) [2], [5]. Despite these advances, it is still not clear which mediator(s) play a central role in disease onset and/or progression of colitis.

As TNF-α and adherent and goblet cell mucin are two major components that are altered in UC, we reasoned that both of these components play major roles in epithelial barrier function and may be selectively altered prior to epithelial cell damage in DSS-induced colitis. In the present study, we used a protocol-treating animal for 9 consecutive days with DSS to characterize the earliest events in disease onset and progression to acute colitis. In particular, we focused on the contribution of TNF-α and colonic mucin in innate host defense prior to epithelial cell damage and investigated whether TNF-α neutralizing antibody can alter disease onset and/or progression in DSS-induced colitis in rats.

## Results

### Disease Activity Index (DAI) and Tissue damage

**Figure 1:**
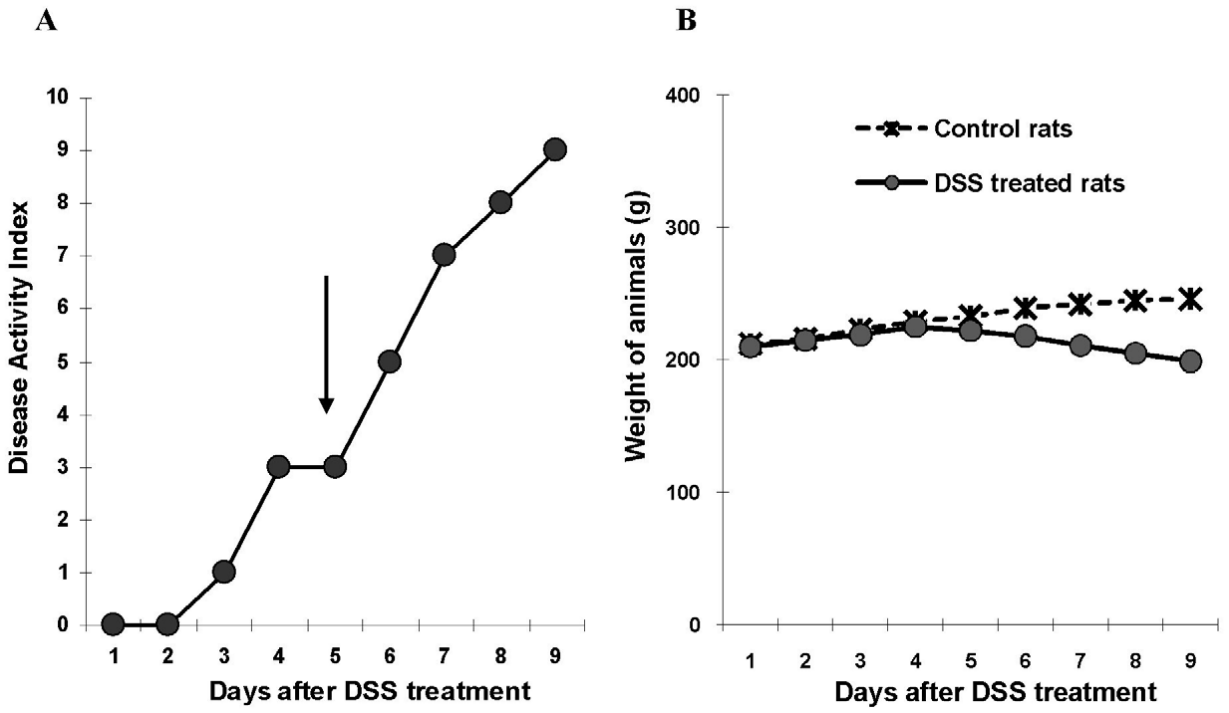
Disease Activity Index (A, DAI) and change in body weight (B) during the progressive development of DSS-induced colitis in rats. Animals received 5% DSS in drinking water for 1–9 days. Note, a striking difference in DAI (*A*) was observed from day 5 onwards (*arrow*). Changes in the body weight (*B*) of control (*asterisk*) and DSS treated animals (*circles*). Loss in the body weight coincides well with increase in DAI. Data represents the means ± SEM from 6 animals per day.

DAI is a cumulative index of body weight loss, rectal bleeding and stool malformation and is considered as the best measure of clinical activity of colitis [6]. DAI during the first 4 days was not associated with significant change in the body weight of control and DSS treated animals. However, on day 5 there was an exponential increase in DAI that continued up to day 9 (Fig. 1A). Clinically, day 5 of DSS treatment was a critical turning point as DAI strongly correlated with weight loss (Fig. 1B) in comparison to control animals. An approximate reduction of 18–20% total body weight was observed in DSS treated animals on day 9. After 9 days of continuous 5% DSS treatment, animals suffered severe rectal bleeding (30%) and/or deaths (10% of animals).

**Figure 2:**
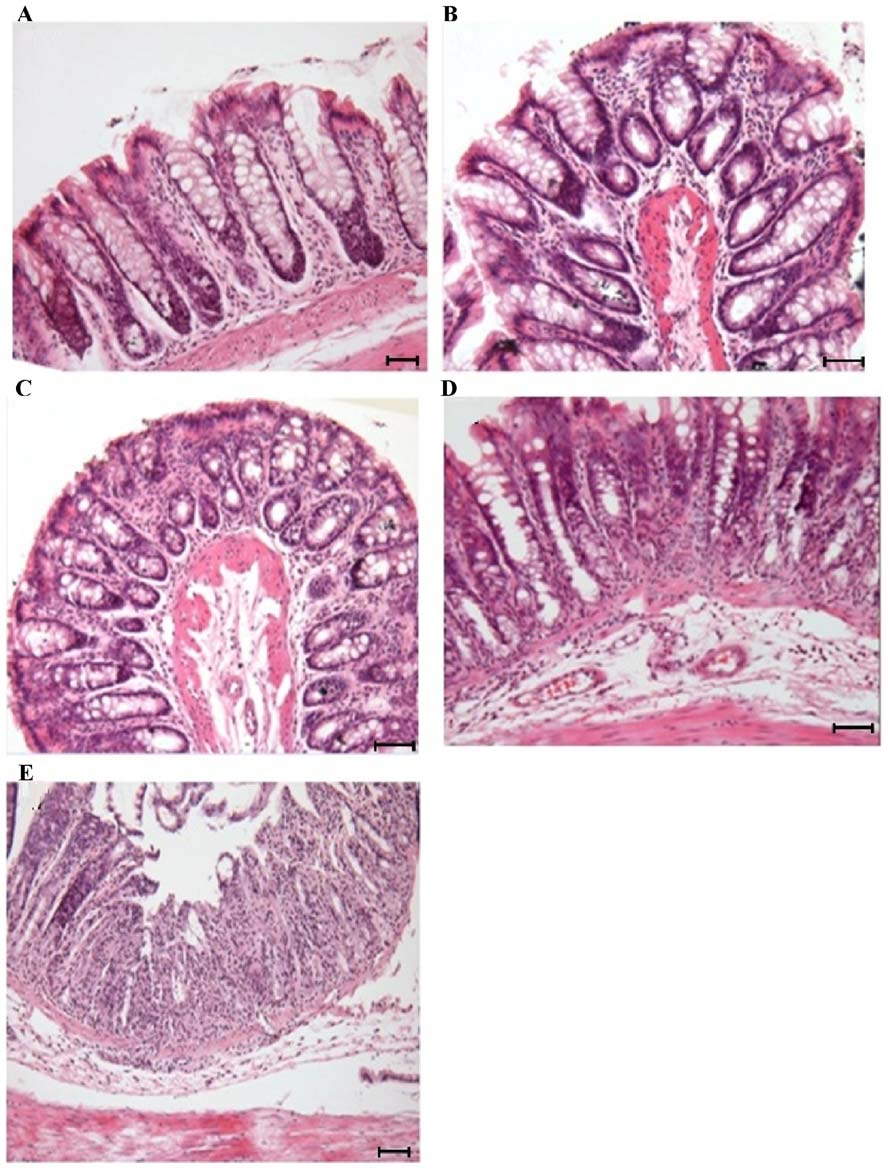
Histopathological characterization of DSS-induced colitis. *A*: Normal control rat colon on day 9 showing well organized crypts and lamina propria and submucosal structures. *B*: DSS treatment on day 2 showing intact mucosal and sub-mucosal structures. *C*: DSS treatment on day 5 showing focal erosions of the epithelium with an acute inflammatory infiltrate. *D*: DSS treatment on day 7 showing crypt dysplasia and edema of the submucosa. *E*: DSS treatment on day 9 showing complete denuding of the surface epithelium, dense cellular inflammatory infiltrate in the lamina propria and loss of crypt structures (Scale bar represents 25 µm; all sections were stained with H&E).

To determine the earliest histological alterations during DSS-induced colitis, serial sections of the distal colon predicted to develop ulcers were evaluated on a daily basis. On days 2–4, no histological alterations were observed (Fig. 2B), but as early as day 5, focal erosions of the epithelium with acute inflammatory infiltrate (Fig. 2C) including lymphocyte and polymorphonuclear lymphocytes were seen in DSS treated animals. In particular, crypt dysplasia (Fig. 2D) was evident during the development of colitis from days 5–9. Notably, moderate to severe submucosal edema, hyperemia and erosions were observed in the colon in DSS treated animals on day 9 (Fig. 2E). Typical histological alteration in the mucosa resembled active UC with severe mucosal and submucosal lesions.

### DSS Alters Colonic Mucin and Muc2/3 Expression

**Figure 3:**
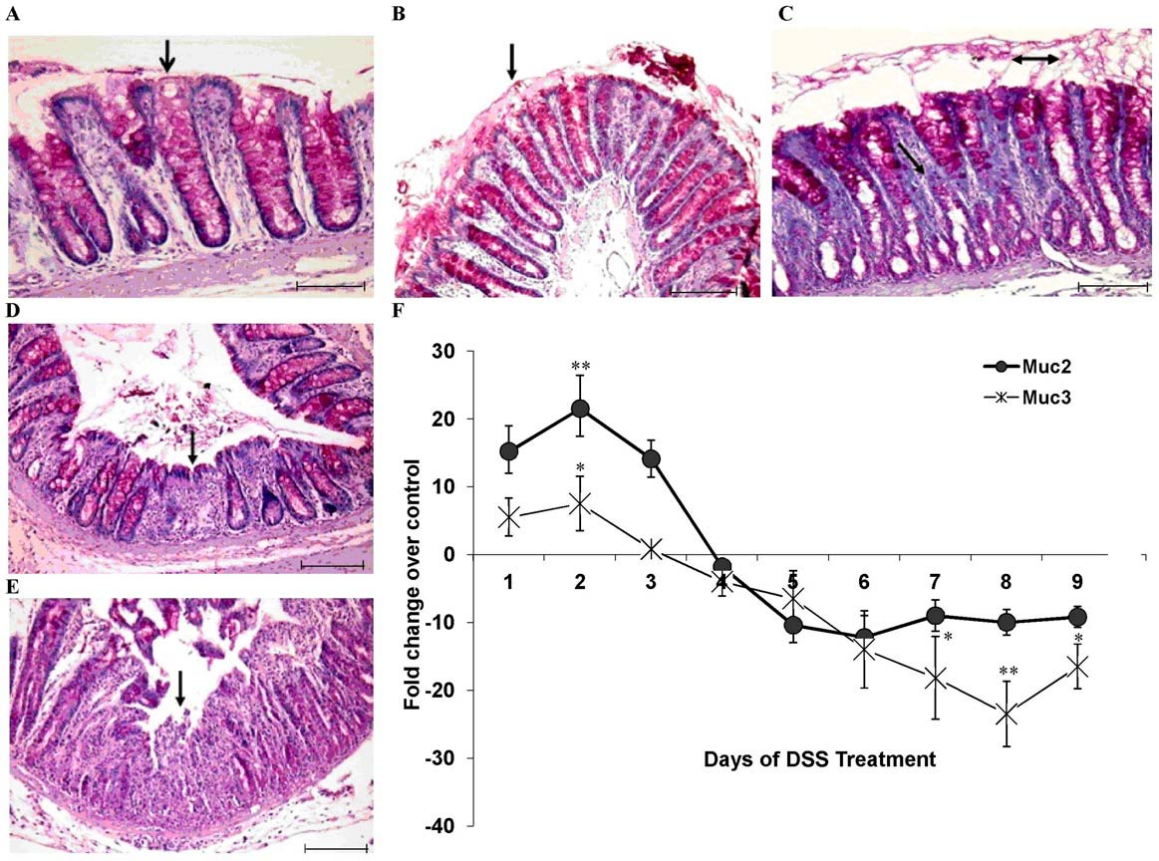
The effect of DSS treatment on adherent and goblet cell mucin content and mucin gene expression. Colonic tissues sections were stained with Periodic acid Schiff reagent to visualize adherent and goblet cell mucin content. *A*: Control rat colon on day 0 showing goblet cells with high mucin content from the base to the tip of the crypts (magenta color). *B*: DSS treatment on day 2 showing mucin filled goblet cells as well as large amount of secreted adherent mucus (single head arrow) in the lumen. *C*: DSS treatment on day 5 showing disrupted elongated basal crypts with little mucin. Goblet cells at the tips of the crypts show intense mucus secretagogue activity with loose disorganized luminal mucus (double head arrow). *D*: DSS treatment on day 7 demonstrating loss of goblet cells at the site of ulcer formation (single head arrow), dense cellular infiltrate and loss of the adherent mucus barrier. *E*: DSS treated rat on day 9 showing ruptured mucosa (single head arrow) with no evident of goblet cells at the site of damage or in the adjacent areas. Scale bar represents 25 µm. *F*: The effect of DSS treatment on Muc2 and Muc3 gene expression. The relative gene expression levels were determined by real time PCR for using mRNA extracted from control and DSS treated rats during the 9 consecutive days of DSS treatment. Expression levels were normalized using GAPDH as housekeeping gene and the mRNA levels plotted as fold change over control. Data shown are the means ± SEM of 6 animals/day. *P<0.05 and **P<0.001 compared to control colon.

Mucin is the first line of host defense and its alteration severely affects epithelial barrier function [7]. Defects in mucosal cell barrier function are related to permeability to macromolecules, increased bacterial invasion and/or translocation which primarily depends upon depletion of the thick viscous mucin layer due to severe mucus secretagogues activity, depletion of mucin by goblet cells and mucin wash out due to mucosal inflammation and diarrhea [8]. As loss of the protective mucus barrier and goblet cell mucin may be the initial inciting event that underlies injury and inflammation in UC, we quantified randomly the number of goblet cells in the crypts that were filled/empty or releasing mucin in DSS-treated rats. In control animals there was a thick adherent mucus layer on the epithelium and well-organized long crypts with dense mucin-filled goblet cells (Fig. 3A). Morphologically, in control animals, 84% of goblet cells were filled with mucin and only 4% of empty goblet cells were seen. However, in DSS-treated animals a significant temporal change in the number and morphology of mucin secreting activity of goblet cells were observed. On day 2 of DSS treatment, goblet cells were filled with mucin (87%) accompanied by a thick adherent and loose mucus exudate in the lumen in the absence of tissue injury or abnormal cellular infiltrate (Fig. 3B and Table 1). However, on day 5, intense mucus secretagogue activity resulted in goblet cells depleted of mucin and in other areas mucus streaming for goblet cells with a thick none adherent mucus layer on the surface epithelium (Fig. 3C). As shown in Table 1, there was a significant decrease in the number of filled goblet cells (21% in comparison to 84% of control) with a corresponding rise in number of empty goblet cells (49% in comparison to 4% of control) and goblet cells releasing mucin (31% in comparison to 12% of control). The mucus cap was layered on the injured surface focal lesions. A curious finding was that goblet cells in the lower half of the crypts were devoid of mucin (Fig. 3C). This time point of high mucin secretagogue activity also coincided with a sharp increase in DAI (day 5). On day 7 of DSS treatment, few goblet cells were seen at the site of well-developed ulcers formation, the mucus cap was completely lost and goblet cells in areas adjacent to the ulcers had very little mucin (Fig. 3D). In particular, there was a significant increase in the number of empty goblet cells (77% in comparison to 4% of control, Table 1). On the day 9 of DSS treatment, goblet cells were almost absent with a paucity of PAS positive proteins in the ulcerated site. In the adjacent areas to the ulcers, crypts were damaged and the few goblet cells contained insignificant amount of mucin (Fig. 3E).

**Table 1:** Morphology of goblet cells from rat tissue treated with DSS for different time point.

	Percentage of total goblet cells		
DSS Treatment	Filled	Releasing Mucus	Empty
Control	84.0±1.3	12.0±0.4	4.0±0.2
Day 2	86.5±0.5	10.4±0.4	3.1±0.5
Day 5	20.9±0.6***	30.5±0.5*	48.6±0.9**
Day 7	17.1±0.64***	5.7±0.4	77.1±1.4***
Day 9	ND	ND	ND

Data are presented as % of goblet cells ± SEM. *P<0.05, **P<0.01, ***P<0.0001 compared to control group. Goblet cell morphology was quantified from randomly selected crypts of PAS stained sections. A minimum number of 100 (100–110) goblet cells were counted under 40 X magnification from each section. Goblet cell morphology was designated as adapted as previously described [28]. Filled goblet cells: goblet cells with intact mucus granules; Releasing mucus: cells releasing mucus with PAS-stained mucus emerging as a thick stream; Empty goblet cells with PAS stained mucus absent from cells exhibiting a deep concave cavitation of the apical surface. ND: Not determined as the epithelial layer and crypts on day 9 DSS treatment was destroyed or too damaged in the ulcerated site.

As a decrease in luminal mucin content may reflect a differential expression of mucin genes, we determine whether the expression of secretory (Muc2) and membrane bound (Muc3) mucin were also altered during the onset of ulcer formation. As shown in Fig. 3F, following a significant increase in Muc2 (∼22 fold) and Muc3 (∼8 fold) gene expression between days 1–2, there was a marked down regulation of Muc2/3 from day 4 onwards (15- and 18-fold lower respectively). No significant difference in the expression of Muc1 was observed in DSS treated and control animals.

### DSS Treatment Alters the Expression Pattern of TLRs

**Figure 4:**
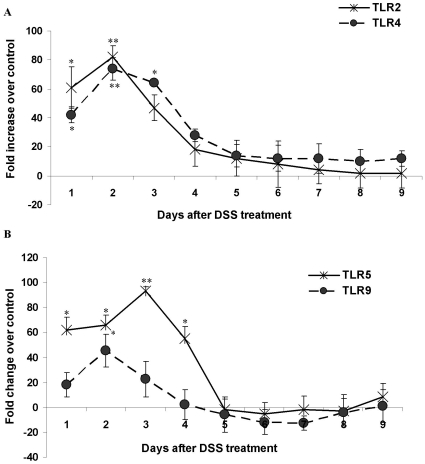
The effect of DSS treatment on the TLR gene expression. Relative gene expression of (*A*) TLR2 (asterisks) and TLR4 (circles) and (*B*) TLR5 (asterisks) and TLR9 (circles) genes in DSS treated rats over controls during 9 days of DSS treatment. The expression levels of TLR genes were determined by real time PCR and normalized using GAPDH as housekeeping gene. The mRNA levels are plotted as fold change over control. Data shown are the means ± SEM of 6 animals/day. *P<0.05 and **P<0.001 compared to control colon.

TLRs are sensors on epithelial cells/APCs that identify and respond to microbes by eliciting effector, regulatory or cytoprotective responses [7]. As changes in TLR expression pattern are critical for the induction of both mucosal effector and regulatory cell responses, the expression of TLR2, TLR4, TLR5 and TLR9 genes involved in microbial recognition was examined. Surprisingly, the expression profiles for TLR2/4 (Fig. 4A) and TLR5/9 (Fig. 4B) genes in DSS treated animals increased 60–80-fold and 10–20-fold, respectively, on days 1–3. The increase in TLR2/4 expression was evidenced through all 9 days of DSS treatment, albeit was not as prominent as during the first three days (Fig. 4A). TLR5/9 expression levels returned to normal on day 5 and remained low up to day 9 (Fig. 4B). DSS was a potent inflammatory insult for TLR expression in the onset of disease.

### DSS Stimulates the Production of Pro-inflammatory and Regulatory Cytokines

**Figure 5:**
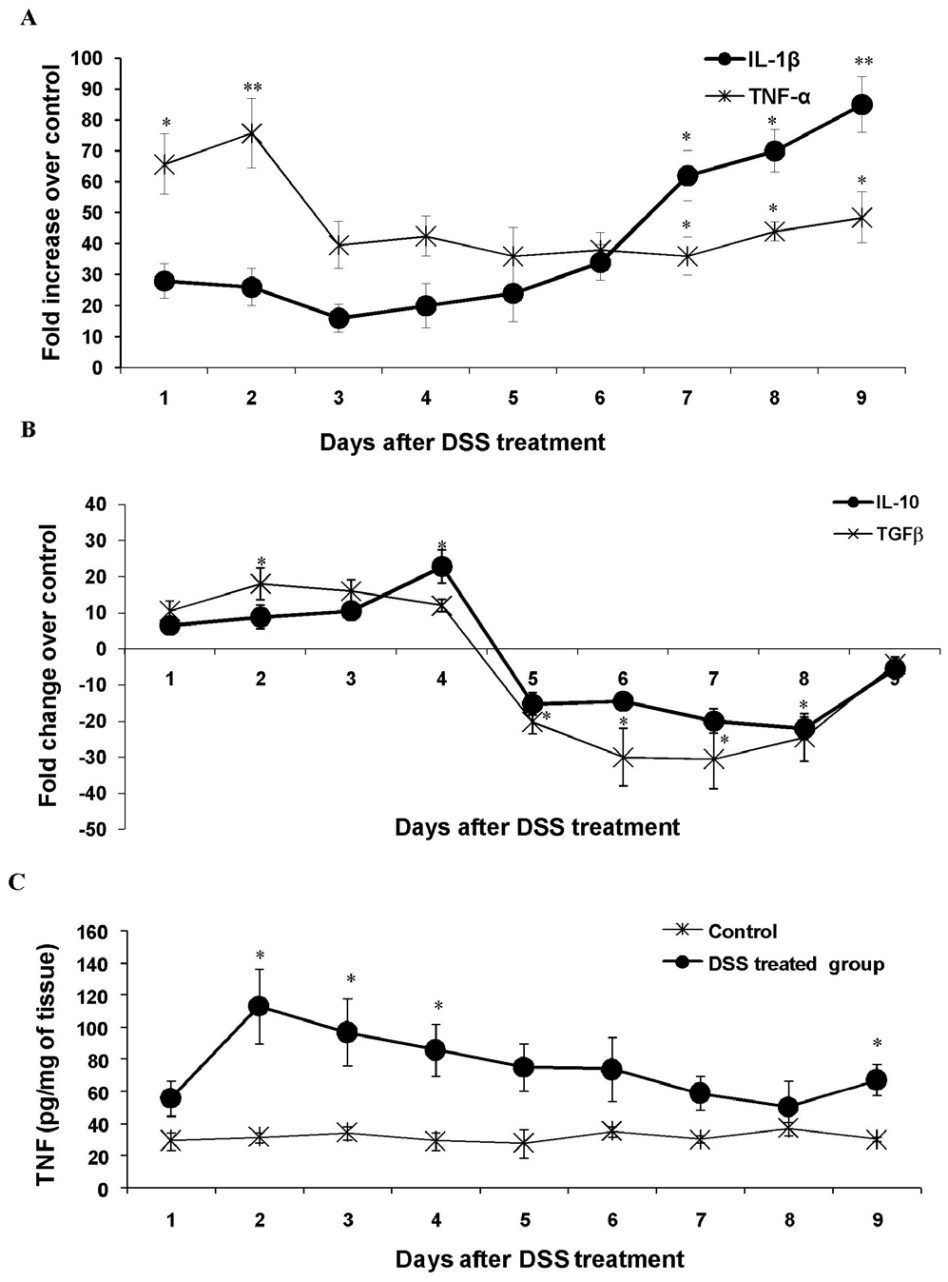
The effect of DSS treatment on pro-inflammatory and regulatory cytokine gene expression. The relative gene expression levels were determined by real time PCR for (*A*) TNF-α (circle) and IL-1β (asterisks) and (*B*) TGFβ (circle) and IL-10 (asterisks) genes using mRNA extracted from control and DSS treated rats during 9 days of DSS treatment. Expression levels of all genes were normalized using GAPDH as housekeeping gene. The mRNA levels are plotted as fold change over control. Data shown are the means ± SEM of 6 animals/day. *P<0.05 and **P<0.001 compared to control colon. *C*: The effect of DSS treatment on the TNF-α protein secretion as measured by ELISA. TNF-α protein in DSS treated rats (circle) and controls (asterisks) are plotted as pg/mg of tissue. Data shown are the means ± SEM of 6 animals/day. *P<0.05 and **P<0.001 compared to control colon.

A break in tolerance to enteric bacteria and an aberrant response to normal luminal flora leading to an immunological imbalance is the hallmark of UC pathogenesis. This imbalance represents both effector and regulatory mucosal immune responses. We therefore next considered the impact of DSS treatment on production of the important pro-inflammatory (TNF-α and IL-1β, Fig. 5A) and regulatory cytokines (IL-10 and TGFβ, Fig. 5B). Pro-inflammatory cytokine expression exhibited a bimodal expression profile (Fig. 5A), which was initially led by a significant increase in TNF-α (∼70–80-fold increase on days 1–2), while acute disease was dominated by significant high expression of IL-1β (∼60–90-fold increase on days 6–9). Even though TNF-α expression decreased to 30-fold on day 3, its expression remained significantly high up to day 9 (Fig. 5A). Predictable, colonic tissues also showed high levels of TNF-α protein (Fig. 5C) and mirrored the TNF-α gene expression profile. Interestingly, the regulatory cytokines IL-10 and TGFβ were significant up regulated during the onset of disease (days 2–4) but at day 5, there was a sharp decline in the expression of both regulatory cytokines that stayed low up to day 9 (Fig. 5B). Early onset and acute colitis was dominated by a marked up-regulation of TNF-α, whereas, IL-1β was prominent in acute disease.

### DSS Treatment Affected MPO Activity and Chemokine Expression

**Figure 6:**
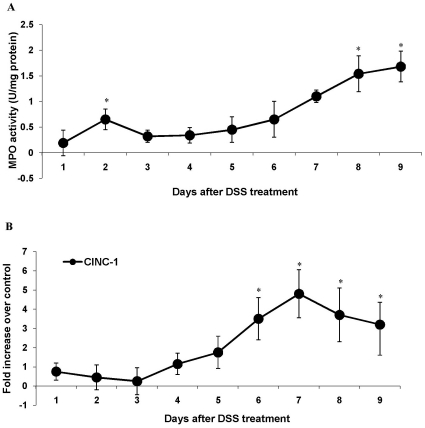
The effect of DSS treatment on the expression and activity of pro-inflammatory mediators. *A*: Myeloperoxidase activity (MPO) was measured in the colonic mucosa of rats administered 5% DSS in drinking water for 1–9 days. MPO activity is expressed as unit per mg tissue and all values are the means ± SEM of 6 animals/day. *P<0.05 compared with normal control groups. *B*: The effect of DSS treatment on CINC expressions. The relative gene expression levels were determined by real time PCR for CINC gene using mRNA extracted from control and DSS treated rats during 9 days of DSS treatment. Expression levels of all genes were normalized using GAPDH as housekeeping gene. The mRNA levels are plotted as fold change over control. Data shown are the means ± SEM of 6-animals/day. *P<0.05 and **P<0.001 compared to control colon.

Colonic myeloperoxidase (MPO) activity is an indicator of neutrophil infiltration and inflammation. DSS treated animals showed a significant rise in MPO activity from day 6 onwards that peaked on day 8–9 (Fig. 6A). This rise in MPO activity during later stages of DSS-induced colitis were further corroborated by the alteration in the gene expression of CINC-1, an analogue of human IL-8 and a rat chemokine that has potent chemo attractant effects on neutrophils [9]. Gene expression analysis of CINC-1 depicted a baseline profile during the onset of disease (days 1–5) but between days 6–9 of acute phase of the disease, a 4–5-fold increase in expression of CINC-1 was observed (Fig. 6B). As expected, MPO activity and CINC-1 expression were highly correlated with DAI.

### The Effect of TNF-α Neutralizing Antibody on Disease Onset and Progression

**Figure 7:**
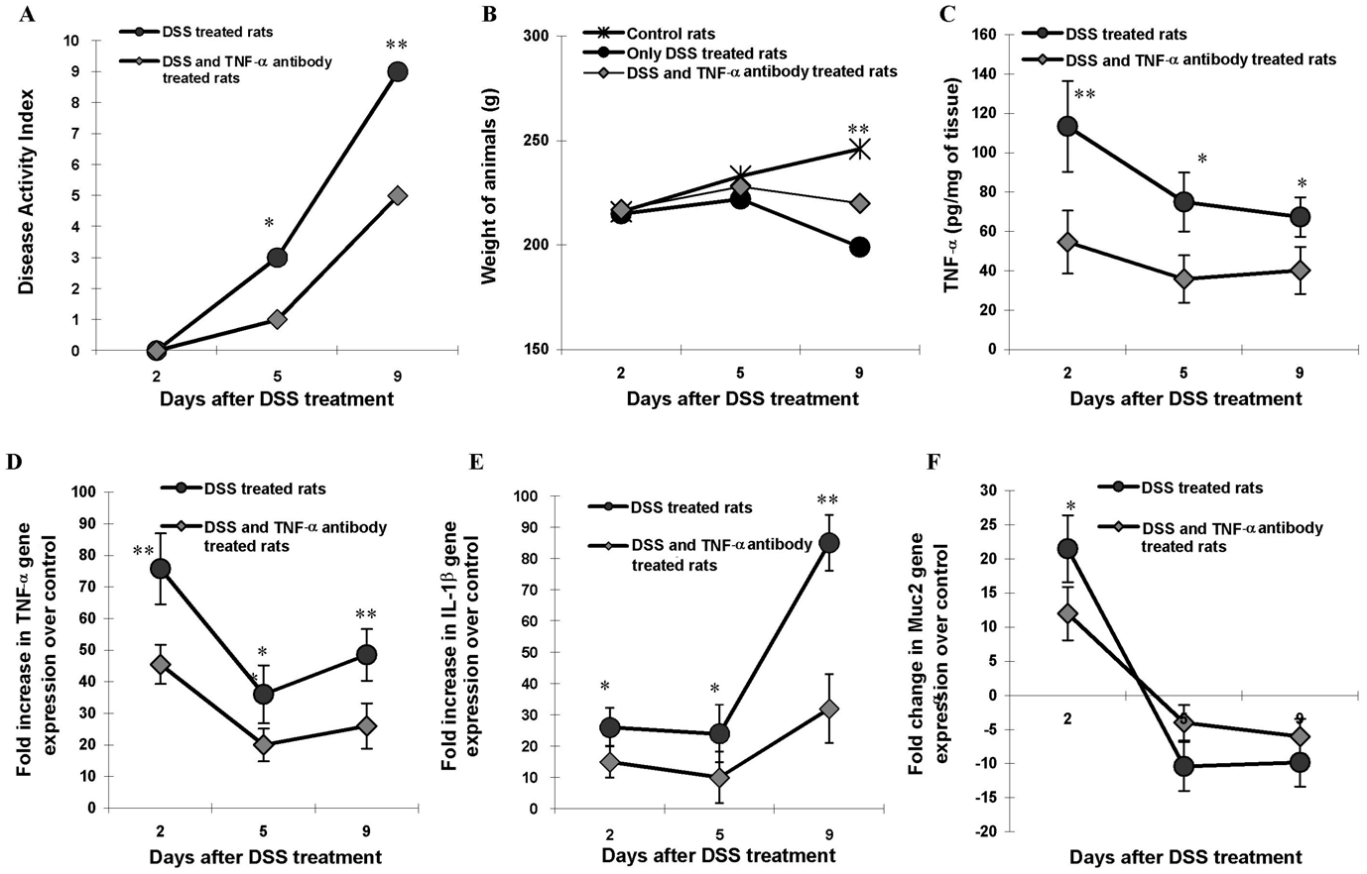
The effect of TNF-α neutralizing antibody treatment on disease onset and progression in DSS induced colitis. *A*: Comparison of disease activity index (DAI) on day 2, 5 and 9 of DSS treatment in animals that received 5% DSS in drinking water alone (black circles) or with TNF-α neutralizing antibody (grey diamonds). *B*: Body weight change plotted for control (asterisks), DSS only (black circles) or DSS + TNF-α neutralizing antibody treatment (grey diamonds). Data represents the means ± SEM from 6 animals per day. *C*: TNF-α protein secretion measured by ELISA on days 2, 5 and 9 of DSS treatment in animals that received 5% DSS in drinking water alone (black circles) or with TNF-α neutralizing antibody (grey diamonds). Data shown are the means ± SEM of 6-animals/day. *P<0.05 and **P<0.001 compared to control colon. *D–F*: The relative gene expression levels determined by real time PCR for TNF-α (*D*), IL-1β (*E*) and Muc2 (*F*) genes on day 2, 5 and 9 of DSS treatment in animals that received 5% DSS in drinking water alone (black circles) or with TNF-α neutralizing antibody (grey diamonds). The expression levels of all genes were normalized using GAPDH as housekeeping gene and mRNA levels are as fold change over control. Data shown are the means ± SEM of 6-animals/day. *P<0.05 and **P<0.001 compared to control colon.

As TNF-α mRNA expression and colonic tissue protein were markedly up regulated in disease onset (Days 2–3, Fig. 5) associated with alterations in Muc2 expression (Fig. 3F) which preceded goblet and luminal mucin alterations, we determined if TNF-α neutralizing antibody could alter the course of the disease. As shown in Fig. 7A, TNF-α neutralizing antibody significant decreased DAI on day 5 and 9 as compared to untreated controls. Notably, the TNF-α neutralizing antibody treated group had significantly lower DAI (Fig. 7A) and higher body weight (Fig. 7B) at the critical day 5 time point associated with the exponential rise in the DAI observed in the DSS treated group. As expected, there was a significant reduction in the levels of TNF-α protein in colonic tissues on days 2, 5 and 9 in the TNF-α neutralizing antibody treated groups as compared to DSS untreated treated animals (Fig. 7C). Consistent with the protein level, TNF-α neutralizing antibody treatment significantly inhibited the up regulation of TNF-α mRNA expression seen in the DSS treated rats (Fig. 7D). The most prominent effect of TNF-α neutralizing antibody treatment was observed on the expression of IL-1β gene expression. IL-1β gene expression was 32-fold higher on day 9 in the TNF-α neutralizing antibody treated group compared to controls (Fig. 7E). In comparison, DSS-treated rats showed 85-fold increase in IL-1β gene expression on day 9 of DSS treatment as compared to controls (Fig. 7E). These results suggest a TNF-α dependent cytokine network in the pathogenesis of DSS-induced colitis.

**Figure 8:**
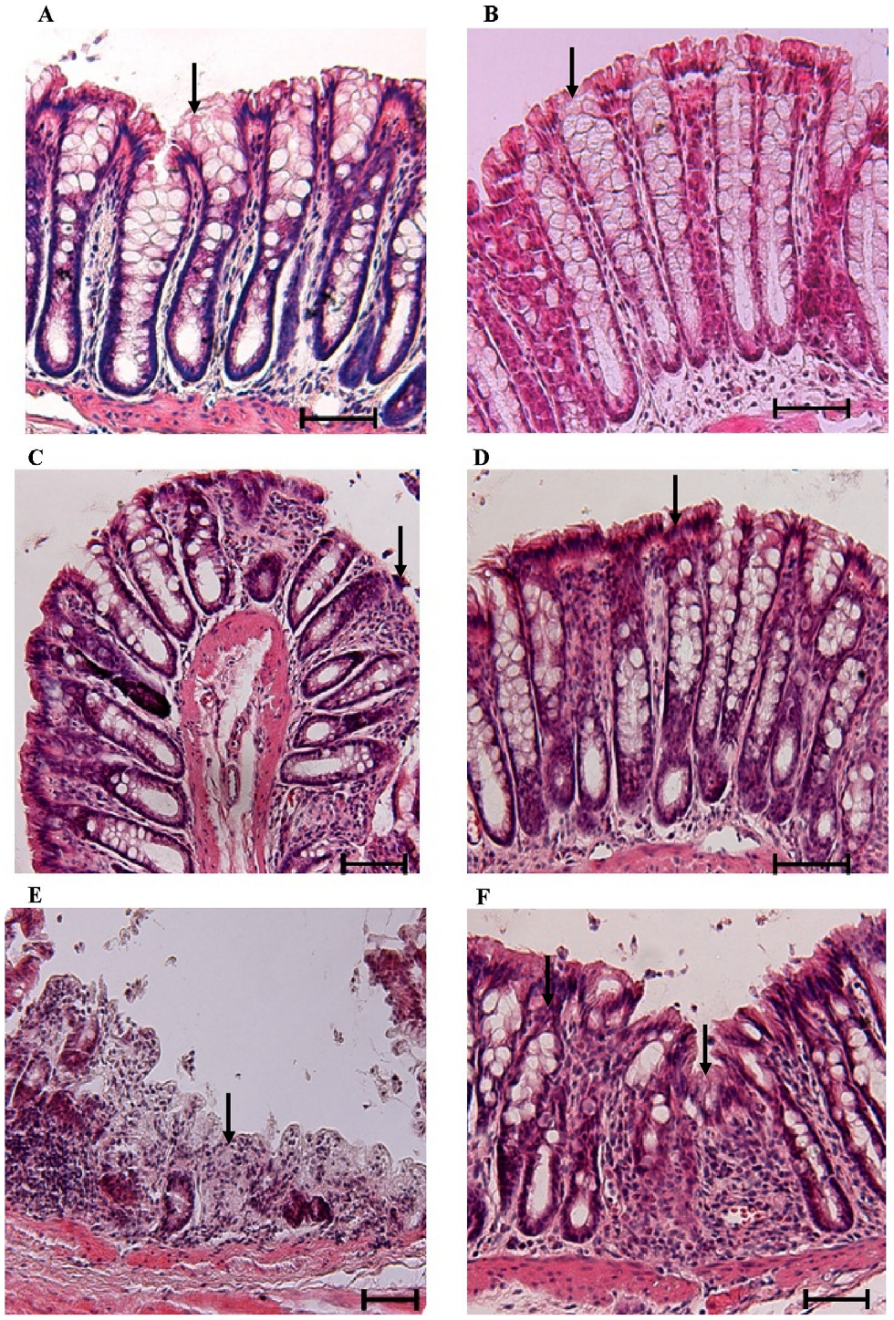
The effect of TNF-α neutralizing antibody on the development of colonic lesions in DSS treated rats. *A*: DSS treatment alone on day 2 showing intact mucosal (arrow) and sub-mucosal structures. *B*: TNF-α neutralizing antibody plus DSS treatment on day 2 showing intact crypts and mucosal (arrow) and submucosal structure. *C*: DSS treatment alone on day 5 showing focal erosions of the epithelium (arrow) with acute inflammatory infiltrate. *D*: The effect of TNF-α neutralizing antibody plus DSS treatment on day 5 showing well developed crypts with no abnormal cellular infiltrate with intact mucosa (arrow). *E*: DSS treatment alone on day 9 showing extensive mucosal damage and deep ulceration (arrow) with large numbers of inflammatory cellular infiltrates. *F*: The effect of TNF-α neutralizing antibody plus DSS treatment on day 9 showing focal erosions (arrow) with less damaged and cellular infiltrate in the mucosal architecture adjacent to the lesion. (All sections were stained with H&E; scale bar represents 25 µm).

**Figure 9:**
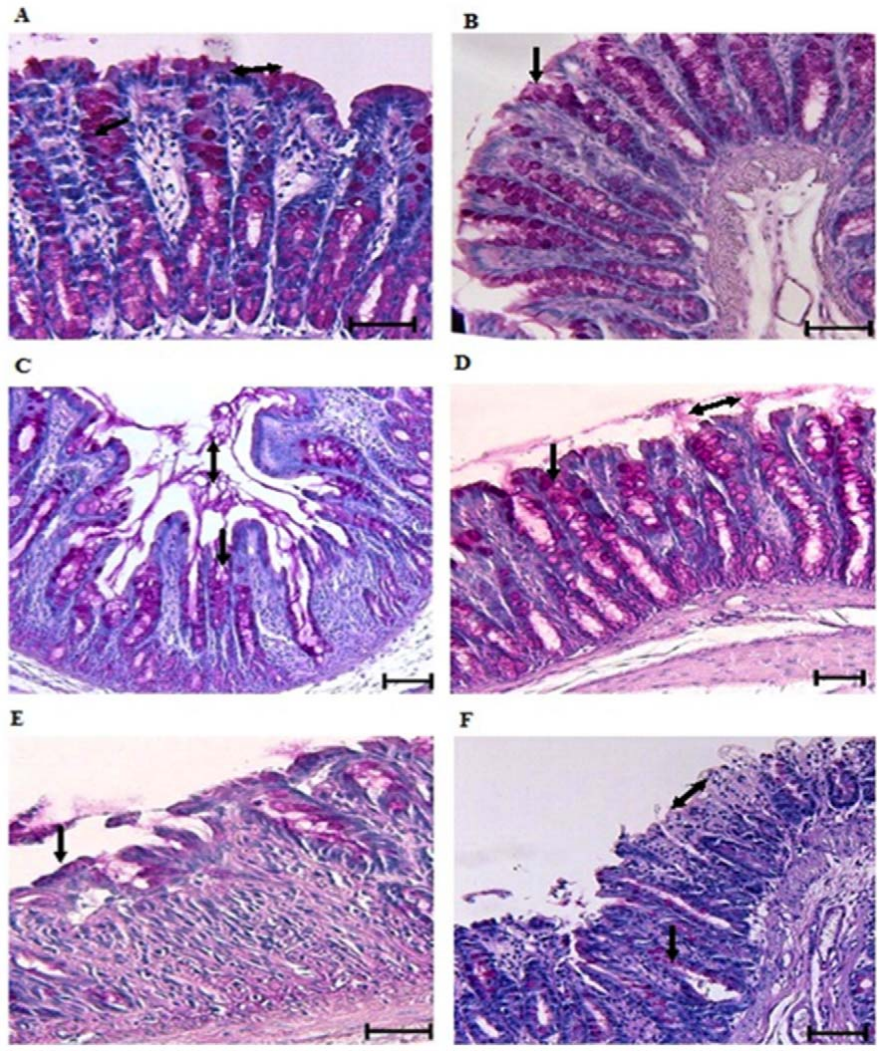
The effect of TNF-α neutralizing antibody on the adherent mucus layer and goblet cell mucin in DSS-induced colitis. Rat colonic tissues sections were stained with Periodic acid Schiff reagent to visualize adherent and goblet cell mucin content. *A*: DSS treatment alone on day 2 showing mucin filled goblet cells (magenta color) and secreted mucin in the lumen (double head arrow) with a modest inflammatory infiltrate. *B*: TNF-α neutralizing antibody plus DSS treatment on day 2, showing mucus secretion from crypt goblet cells. *C*: DSS treatment only rat on day 5 showing disrupted crypts, intense inflammatory cellular infiltrate and intense mucus secretion from crypt goblet cells (single head arrow) with loss of the adherent mucus layer (double head arrow). *D*: TNF-α neutralizing antibody plus DSS treatment on day 5 showing well organized crypts filled with mucin (single head arrow) and an adherent mucus layer (double head arrow) on the surface epithelium. *E*: DSS treatment on day 9 showing an ulcerated region with a ruptured mucosa with complete loss of crypts. The residual goblet cells adjacent to the ulcer show remnants of mucin. *F*: TNF-α neutralizing antibody plus DSS treatment on day 9 demonstrating only to the surface mucosa (double head arrow) and the deep crypts show evidence of mucin in goblet cells beneath the lesions (single head arrow). Scale bar represents 25 µm.

In addition to the effect on pro-inflammatory cytokine production, TNF-α neutralizing antibody also showed significant cessation in the alteration of mucin expression and mucus production triggered by DSS-treatment. Muc2 gene expression was only 12-fold higher on day 2 and 6-fold lower on day 9 in the TNF-α neutralizing antibody treated group as compared to controls (Fig. 7F). In comparison, DSS-treated rats showed a 22-fold increase in the Muc2 gene expression on day 2 and a 10-fold decrease on day 9 when compared to controls. H&E and PAS staining showed less focal erosions and inflammatory cellular infiltrate (Fig. 8 and Fig. 9), more adherent mucus in the lumen and organized long crypts containing mucin-filled goblet cells on day 2 and 5 in the TNF-α neutralizing antibody treated group. Even on day 9, mucins filled goblet cells were seen in the colonic tissue of the TNF-α neutralizing antibody treated group whereas untreated DSS treated rats showed mucin-devoid goblet cells from day 5 onwards (Fig. 9F). In particular, on day 5 there were more filled goblet cells (60% in comparison to 37% of DSS treated group) and less numbers of empty goblet cells (29% in comparison to 40% of DSS treated group) in the TNF-α neutralizing antibody treated group compared to the DSS only treated group (Table 2). Even on day 9, there were goblet cells in ulcerated area, whereas in the DSS only treated group we were no goblet cells in the ulcerated areas. The ulcerated areas in the TNF-α neutralizing antibody treated group on day 9 seem restricted to the surface epithelium (Fig. 9F) with well-organized crypts with mucin filled goblet cells. These data suggest that neutralizing TNF-α markedly affected mucin release, mucus depletion and crypt inflammation to restrict the mucosal damaging effects of DSS.

**Table 2:** Goblet cell morphology in DSS + TNF-α neutralizing antibody treated group in comparison to only DSS treatment.

Groups		Percentage of total goblet cells		
		Filled	Releasing Mucus	Empty
Day 2	DSS only	77.4±3.6	16.5±1.2	6.1±0.6
	DSS + anti-TNF-α	67.9±1.2	22±0.7	10.1±0.5
Day 5	DSS only	37.1±0.9	22.6±0.5	40.2±0.7
	DSS + anti-TNF-α	59.6±1.3^*^	22.8±0.7	29.4±1.6^*^
Day 9	DSS only	ND	ND	ND
	DSS + anti-TNF-α	19.6±0.4	21.6±0.3	58.8±1.0

Data are presented as % of goblet cells ± SEM. *P<0.05 when compared to DSS treated group of the respective day. Goblet cell morphology was quantified from randomly selected crypts of PAS stained sections. A minimum number of 100 (100–110) goblet cells were counted under 40 X magnification from each section. Only in the DSS + anti-TNFα treated group we were able to count 51 goblet cells. Goblet cell morphology was designated as previously described [28]. Filled goblet cells: goblet cells with intact mucus granules; Releasing mucus: cells releasing mucus with PAS-stained mucus emerging as a thick stream; Empty goblet cells with PAS stained mucus absent from cells exhibiting a deep concave cavitation of the apical surface. ND: Not determined as the epithelial layer and crypts on day 9 of DSS treatment was destroyed or too damaged in the ulcerated site.

## Discussion

This is the first comprehensive study to quantify the salient features of early onset and acute progressive events in the pathogenesis of DSS induced colitis. Onset of disease (days 1–5) was characterized by elevated levels of TNF-α mRNA expression, protein production, depletion of luminal adherent mucin and goblet cell mucin stores prior to the appearance of focal erosions on mucosal epithelial cells. These early events resulted in a progressive increase in DAI (from day 5 onwards) and weight loss associated with rectal bleeding and organized ulcers in the distal colon. Treatment with TNF-α neutralizing antibody significantly decreased DAI, delayed the acute phase of colitis and effectively curtailed alterations in the expression and production of TNF-α and mucins. These results suggest that both increased TNF-α and mucin depletion was a prerequisite for the development of focal erosions. The acute phase of disease was dominated by loss of luminal and cellular mucin stores, down regulation of regulatory cytokines, elevated levels of MPO, CINC-1 and IL-1β expression.

The most dominant biomarker in the onset of disease was the pro-inflammatory cytokine-TNF-α, which was increased 70–80-fold suggesting that it played a major role in innate host defense. TNF-α is a 17-kda pro-inflammatory cytokine produced by monocytes, macrophages, and T cells. Our data suggests that TNF-α target epithelial cells (and perhaps lymphocytes) during the initial phase of colitis to trigger a cytokine network as well as to enhance mucin production. Treatment with TNF-α neutralizing antibody significantly reduced both DAI and IL-1β and Muc2 gene expression induced by DSS treatment. Our findings are in contrast with a report that shows an exacerbated DSS-induced colitis in TNF-α knockout mice [10]. We speculate that this might be due to the partial blockage of TNF-α achieved with TNF-α neutralizing antibody in our study and that complete knocking out of the TNF-α gene may have triggered other pro-inflammatory responses [10]. Another critical characteristic of this phase is high mucin content in goblet cells and a significant up regulation Muc2 and Muc3 gene expression. This is contradictory to the view that impairment of mucosal barriers via depletion of mucin layer and/or downregulation of mucin producing genes may be an early event of pathogenesis. The up regulation of mucin genes could also be an early event of inflammation triggered by pro-inflammatory cytokines including TNF-α [11]. Perhaps mucin production is a component of the inflammatory responses of epithelial tissue [12], [13]. Several studies have shown that mucin secretion is increased upon IL-1β, IL-4, IL-13 or TNF-α stimulation [14]–[16]. Other noteworthy changes during the first 1-3 days are an extensive up regulation of TLR2 and 4 and controlled up regulation of TLR5 and TLR9. *In vivo* and *in vitro* studies have shown an exaggerated TLR expression (especially TLR4) that leads to an uncontrolled immune response (Th1 or Th17 mediated) against resident microflora [17]. It could be noted that no significant change in DAI or histological damage of colonic mucosa was seen in the early phase of colitis suggesting that the clinical sign of colitis are not evident unless there is complete destruction of mucosal homeostasis. Importantly, this phase with no significant DAI was prolonged in rats treated with TNF-α neutralizing antibody. The pro-inflammatory cytokine TNF-α was the most prominent early biomarker of DSS-induced colitis based on its extensive up regulation during the onset of colitis induction and the fact that treatment of TNF-α neutralizing antibody significantly reduced DSS induced DAI.

Our data suggests that days 4–6 are the most crucial in the induction of DSS induced colitis. Unlike a universal up-regulation of different genes seen in the onset of disease, days 4–6 showed a differential expression profile of various genes suggesting an alteration in factors responsible for mucosal homeostasis. For example, there was exponential rise in DAI (mainly due to extensive drop in the body weight) and the presence of focal lesions from day 5 onwards in DSS animals. Curiously, this phase was dominated by the down regulation of regulatory and cytoprotective factors. In particular, the regulatory cytokines IL-10 and TGFβ were significant down regulated in DSS treatment (40-fold less than controls). Down regulation of regulatory T cell activation including Treg and Th3 cells (secreting TGFβ and IL-10) is a major predisposing factor in the pathogenesis of IBD [18], [19]. Decrease in TGFβ leads to diminished regulation of Th1, Th2 and Th17 effector T cell activation and also more epithelial cell apoptosis, while lowered IL-10 expression leads to more aggressive macrophage activity. An initial upregulation of mucin genes was replaced by sudden down regulation of both Muc2 and Muc3 genes supporting the fact impairment of the intestinal mucosal barrier may lead to high mucosal permeability and diminished epithelial protection [20] that probably leads to later immune assault. A number of reports have documented that inflamed and non-inflamed intestinal tissues in UC and CD have impaired and permeable mucosal barriers [20], [21]. Another critical aspect is the change in the expression profile of various TLR genes. TLR2 and 4 continued to be up regulated though not as extensive as in the first phase, but TLR5 and 9 showed 5–15 fold down regulation from day 5 onward. The results points toward the putative tolerogenic role for the two PRRs. It has been reported earlier that low expression of TLR5 is seen in both forms of IBD [22]. The pro-inflammatory cytokines continued to be up-regulated but in more controlled fashion than the initial phase. Significant delay in reaching the acute phase, relatively intact mucosal architecture, significant mucin content in the goblet cells and limited down regulation of Muc2 gene in TNF-α neutralizing antibody treated rats on day 5 and 9 of DSS treatment further suggests that depletion of mucosal barrier is a key event during transition of initial to acute phase. Together these results reinforces that day 5 of DSS treatment is a critical time point exhibiting a sharp rise in DAI complemented by extensive change of trend for the expression/production of TLR, cytokine and mucin genes. DAI, mucosal depletion and regulatory cytokines, by virtue of their prominent down-regulation appears to be the most prominent and indicative biomarker at this stage in the pathogenesis of DSS-induced colitis.

Acute inflammation on days 7–9 was dominated by an extensive increase in the expression of pro-inflammatory cytokines with IL-1β taking up the center stage (an increase of 80-fold higher expression than controls on day 9) and TNF-α showing second most prominent change in expression (50-fold higher expression than controls on day 9). The results are suggestive of the predominating role of T cells in the later stages of colitis. Genes encoding mucin, IL-10, TGFβ, TLR5 and TLR9 continued to be down regulated during this phase. Colitis was well documented at this stage with the highest DAI score on day 9 and histological analysis showed crypt damage, dysplasia, inflammatory infiltrates and ulcerations in the mucosa of the DSS treated group. In UC, leucocytes numbers are increased associated with high migration from the vasculature into the intestinal mucosa mediated by several chemokines including IL-6, RANTES, MCP1 and MCP2 mediated by various adhesion molecules [23], [24]. This is followed by high release of tissue damaging deleterious metabolites and mediators including nitric oxide, free oxygen radicals, PGs, leukotrienes, histamine, proteases and MMPs by macrophages and other immune cells [25], [26], which cause extensive mucosal damage analogous to what is observed in DSS colitis.

In conclusion, this study demonstrates for the first time that mucosal TNF-α and alteration of the adherent mucus barrier are predisposing factors for early onset epithelial cells damage in DSS colitis. In contrast, high-sustained levels of TNF-α and depletion of adherent and goblet cell mucin are necessary for maintenance of acute colitis. Treatment with TNF-α neutralizing antibody significant altered the onset and severity of disease and prevented the loss of the adherent mucus layer and goblet cell mucin.

## Materials and Methods

### Animals

Six-week-old male Sprague–Dawley rats weighing between 250 and 300 g (Charles River, St. Constant, Quebec) were housed in cages 2 per group at a constant room temperature, with 12-h light and dark cycles, and fed standard rodent chow and water *ad libitum*. Following a 7-day acclimation period, rats were randomized into experimental and control groups for induction of colitis. The Animal Experiment Ethics Committee of the University of Calgary, Canada approved this study (ID MO8123).

### Experimental Design and Induction of Colitis

To study the earliest events in disease onset and progression of DSS induced colitis rats were divided into two groups, controls which had free access to water and the DSS colitis group which had free access to a water containing 5% DSS (wt/vol; molecular weight 50 KDa; Fisher Biotech, Canada) for 9 consecutive days. Animals were sacrificed on all consecutive days of DSS treatment from day 0 to day 9. A total of 18 animals were utilized for each time point (*N* = 6 for each trial/day). To study the effect of anti-TNF-α neutralizing antibody on disease onset and progression of colitis a third group of rats were treated with neutralizing TNF-α antibody in addition to the above-mentioned control and DSS colitis group. The TNF-α antibody treated group were administered the antibody at a dose of 100 µg/animal/day (for rationale, see the section on TNF-α neutralizing antibody treatment) and had free access to water containing 5% DSS for 9 consecutive days. A total of 18 animals were utilized for each time point (*N* = 6 for each trial/day). On the day of sacrifice, animals were given sodium pentobarbital anesthesia (35 mg/kg body weight). Blood specimens were collected by cardiac puncture for flow cytometric enumeration of circulating leukocyte and T cell subset counts. Colons were immediately excised, rinsed with ice-cold phosphate-buffered saline, and placed on ice and four cross sections of the each proximal, middle and distal colon (50–100 mg) were collected. Three cross sections were snap-frozen in liquid nitrogen and stored at −70°C for RNA isolation, protein preparation and analysis of myeloperoxidase activity. The fourth cross section was immediately fixed in 10% neutral buffered formalin for histological analysis.

### Disease Activity Index and Pathological Evaluation of Colitis

Disease Activity Index (DAI) was quantified using the parameters of animal weight loss, stool consistency, and gross blood in the feces, which were recorded daily for each animal. These parameters were each assigned a score, which was utilized to calculate an average daily (DAI) for each animal, as previously described [27].

### Macroscopic and Histological Examination

The proximal, middle and distal colon were examined macroscopically and reported as showing: no mucosal lesions, hyperemia, edema or small area of erosion/ulceration and extensive, marked erosion/ulceration. Assessment of body weight and evaluation of stool consistency (diarrhea) and rectal bleeding were performed on a daily basis. Body weight was assessed at baseline and every day for the duration of the experiment in the control and DSS-treated groups. Weight change was calculated as percentage change in weight compared with baseline. Animals were monitored for rectal bleeding, diarrhea and general signs of morbidity. In three separate preliminary experiments, we consistently observed that most ulcers developed in the distal colon 1–2 cm from the anus. We therefore designated this area for tissues examination for all subsequent experiments listed below.

Tissue sections from the distal colon were fixed in 10% buffered formalin, embedded in paraffin and 6 µm sections were stained with hematoxylin and eosin (H&E) and Periodic acid-Schiff (PAS). Microscopically, H&E tissues were reported as showing: a normal appearance, mild infiltrates of small round cells and polymorphonuclear leukocytes into the lamina propria mucosa, with either no or only shallow erosion, or deep erosion, ulceration and marked infiltration with small round cells and polymorphonuclear leukocytes, often including crypt abscess formation. PAS was used to visualize pre-formed mucin in goblet cells and mucin that were secreted and/or in the mucus gel in the lumen. Mucins in goblet cells were quantified as previously described [28]. Filled goblet cells: goblet cells with intact mucus granules; Releasing mucus: cells releasing mucus with PAS-stained mucus emerging as a thick stream; Empty goblet cells with PAS stained mucus absent from cells exhibiting a deep concave cavitation of the apical surface.

### MPO Activity in Colonic Tissues

For measurement of MPO activity tissues were weighed and homogenized in 10 volume of 50 mM phosphate buffer (pH 6.0) at 4°C, and centrifuged at 30,000× *g* for 30 min at 4°C. The pellet was extracted with 0.5% hexadecyltrimethylammonium bromide (HTAB; Sigma Chemical Co.) in 50 mM phosphate buffer (pH 6.0) at 25°C(23). Samples were sonicated for 10–15 sec and then centrifuged at 30,000× *g* for 30 min. Supernatants were reacted with o-dianisidine dihydrochloride (Sigma Chemical Co.) containing 1 µL/mL of 3% H2O2, and MPO activity was assayed in a 96-well microtiter plate by mixing 20 µl of the supernatant. The change in absorbance was measured spectrophotometrically. Bradford assay was used to measure protein content in the supernatant and results are expressed as MPO activity (mU/mg).

### Quantitative Real-Time PCR Analysis

Quantitative real time PCR analysis was performed to assess changes in the expression of genes encoding for the major secretory and membrane bound mucins (Muc2, Muc1 and Muc3), Toll-like receptors (TLR2, TLR4, TLR5 and TLR9), Cytokine-induced neutrophils chemoattractant (CINC) and various pro-inflammatory (TNF-α and IL-1β) and regulatory (IL-10 and TGFβ) cytokines. Total RNA was extracted with TriZol reagent (Invitrogen). The yield and purity of the RNA was determined by spectroscopic analysis. 2 µg of RNA was reverse transcribed using M-MLV reverse transcriptase (Invitrogen) as per manufacturer's instructions. One microlitre of cDNA was used for real-time PCR (Corbett Research). Real-time primers used with the specific annealing temperatures are shown in Table 3. Amplifications were carried out with Qiagen's Quantitect SYBR Green PCR kit by using the following cycling conditions: 94°C hold for 15 min, followed by 40 cycles of denaturation at 94°C for 20 s, annealing at different temperatures for 30 s and extension at 72°C for 30 s following the manufacturer's instructions. Specificity of amplification was checked by melt curve analysis. mRNA expression for the different genes was normalized against GAPDH and fold change over control was determined according to the ddCt method [29].

**Table 3:** Rat primers sequences and their annealing temperature.

Gene		Primer Sequence	Annealing temperature
Muc1:	Forward	GAGTGAATATCCTACCTACCAC	58°C
	Reverse	TTCACCAGGCTAACGTGGTGAC	
Muc2:	Forward	GCCAGATCCCGAAACCA	55°C
	Reverse	TATAGGAGTCTCGGCAGTCA	
Muc3:	Forward	AACTTCCAGCCCTCCCTAAG	50°C
	Reverse	GCTTCCAGCATCGTCTCTCT	
TLR2:	Forward	GAGTCTGCTGTGCCCTTCTC	50°C
	Reverse	CATGAGGTTCTCCACCCAAT	
TLR4:	Forward	GTTGGATGGAAAAGCCTTGA	50°C
	Reverse	CCTGTGAGGTCGTTGAGGTT	
TLR5:	Forward	GAAGGCTGTGAATCTCGTTGG	50°C
	Reverse	CTGCCCAACCTCAGGATCTTA	
TLR9:	Forward	CCTGGCACACAATGACATTCA	50°C
	Reverse	TAAGGTCCTCCTCGTCCCA	
IL-1β:	Forward	CACCTCTCAAGCAGAGCACAG	59°C
	Reverse	GGGTTCCATGGTGAAGTCAAC	
TNFα:	Forward	AAATGGGCTCCCTCTCATCAGTT	59°C
	Reverse	TCTGCTTGGTGGTTTGCTACGAC	
IL-10	Forward	GGCTCAGCACTGCTATGTTGCC	65°C

### Protein Level of TNF-α in Colonic Tissues

TNF-α in homogenized intestinal tissues was measured using an ELISA kit according to the manufacturer's protocol (rat TNF-α ELISA Kit, Abcam, Canada). Colonic tissues (20 mg) were homogenized in cold phosphate buffer saline (PBS) using a Polytron homogenizer and centrifuged at 20,000 g for 20 min to 4°C to obtain the supernatant. Total protein concentration of the tissue supernatant was measured using a BCA kit. TNF-α is expressed as pg/mg of tissue.

### Assessment of Administration of Anti TNF-α Neutralizing Antibody on the Onset and Progression of DSS-Induced Colitis

In preliminary experiments, three doses of anti TNF-α antibody (50, 100 and 200 µg/animal/day; ip) were administered to animals for 9 consecutive days with free access to water containing 5% DSS. A dose of 100 µg/animal/day was found most optimal in significantly reducing DAI (data not shown) and was used for all the further experiments. DAI was quantified and histological examination was performed for colitis as described above. A quantitative real time PCR analysis was performed using RNA isolated form colonic tissue to assess the changes in the gene expression of TNF-α in addition to other genes (Muc2, TLR4, TLR5, IL-1β, IL-10 and TGFβ) that have shown alteration on DSS treatment in the first set of experiments (see results). TNF-α protein in homogenized intestinal tissues was also measured using ELISA as described above.

### Statistical Analysis

Results are expressed as means ± SD. Significance differences between control and the other strains were determined using Kruskal-Wallis test with Dunns post-test to compare specific groups. The choice of a non-parametric test (Kruskal-Wallis test) instead of a parametric test (Analysis of Variance, ANOVA) was based on the fact that at least one of the groups in all but two of the comparisons was non-Gaussian. To maintain consistency, Kruskal-Wallis test was used for all comparisons. All statistical analyses were performed using Graph Pad Instat software. *P* values >0.05 were considered significant.
